# Infant Passive Protection Against Coronavirus Through Exclusive Breastfeeding: A Cross-Sectional Study

**DOI:** 10.3390/nu17010130

**Published:** 2024-12-31

**Authors:** Yasmin Amaral, Antonio Egidio Nardi, Daniele Marano, Ana Carolina Carioca da Costa, Maria Elisabeth Moreira

**Affiliations:** 1 Postdoctoral Program—Fundação Carlos Chagas Filho de Amparo à Pesquisa do Estado do Rio de Janeiro (FAPERJ), Rio de Janeiro 21941-909, Brazil; 2 National Institute of Women, Children and Adolescents Health Fernandes Figueira-Fiocruz, Rio de Janeiro 22250-020, Brazil; danielemarano@yahoo.com.br (D.M.); carol.carioca@gmail.com (A.C.C.d.C.); bebethiff@gmail.com (M.E.M.); 3 Institute of Psychiatry, Medical School, Federal University of Rio de Janeiro, Rio de Janeiro 21941-617, Brazil; antonioenardi@gmail.com

**Keywords:** vaccine, SARS-CoV-2, breastfeeding, exclusive, antibodies, neutralizing

## Abstract

Background/Objectives: This study aimed to determine the percentage and duration of neutralizing antibodies against the Omicron variant in human milk after vaccination against SARS-CoV-2, considering the three different vaccine technologies approved in Brazil. Methods: A cross-sectional study was conducted with lactating women who received the complete vaccination cycle with available vaccines (AstraZeneca, Pfizer, CoronaVac, and Janssen). The participants resided in Rio de Janeiro, and samples were collected from April to October 2022. Breast milk was analyzed for evidence of neutralizing antibodies using specific assays for the Omicron variant. Results: The results showed that all types of vaccines were effective in inducing neutralizing antibodies in breast milk regardless of the vaccine technology used. There was no significant difference between women receiving two or three doses. Exclusive breastfeeding was significantly associated with higher percentages of neutralizing antibodies in breast milk compared to non-exclusive breastfeeding. Conclusions: These findings reinforce the importance of breastfeeding as a strategy to provide passive protection to infants, especially where vaccination for children under six months of age is not recommended.

## 1. Introduction

Vaccination against COVID-19 is essential to protect the population’s health, preventing severe complications and hospitalizations and reducing morbimortality rates associated with the disease [1]. The prevalence of vaccination against COVID-19 among pregnant women varies between countries. In 2022, two years into the declaration of a public emergency, the CDC found that approximately 69.4% of pregnant women had been vaccinated in the United States by April 2022 [2].

Similarly, in the United Kingdom, studies have indicated that approximately 62% of pregnant women had received at least one dose of the COVID-19 vaccine by the end of 2021 [3,4]. Although global recommendations have endorsed the importance of vaccination since the pandemic’s onset, vaccine hesitancy remains a significant challenge, especially in populations with greater misinformation and lack of access to health services [5].

In Brazil, vaccination coverage among pregnant women varies significantly. In 2021, approximately 50% of pregnant women had been vaccinated with at least one COVID-19 vaccine dose, with higher rates in the southeast, where access to health services is more structured [6]. However, vaccination rates were lower in areas with more limited infrastructure and health services, such as the north and northeast, reflecting the same challenges mentioned above in addition to the difficulties in accessing vaccination points [6].

Exclusive breastfeeding is recommended as the sole source of nutrients for the first six months of life. Complementary foods must be introduced from this period onwards, and breastfeeding must continue for two or more years [7]. Breast milk contains immunocompetent substances (immunoglobulin A (IgA) enzymes, interferon), besides trophic factors or growth modulators [8], that strengthen the newborn’s immune system, ensuring protection against several infections, including COVID-19 [9].

Breastfeeding remained essential for maternal and child health during the COVID-19 pandemic. Studies and guidelines from health organizations, such as the World Health Organization [10] and the Centers for Disease Control and Prevention [11], highlighted the importance of maintaining breastfeeding even amid the health crisis. These recommendations were supported by evidence demonstrating that breast milk does not transmit SARS-CoV-2 and contains immunoglobulins, particularly IgA, which are crucial for protecting infants against infections and supporting the development of their immune systems [9,12].

Evidence suggests that complete and infectious viral particles have not been found, although SARS-CoV-2 RNA can be detected in the milk of infected mothers as isolated fragments. The mammary glands have protective mechanisms that prevent intact and infectious viruses from infiltrating breast milk. So, even if the virus is present in the mother’s blood, it cannot cross these barriers and replicate in breast tissue [12].

Furthermore, some studies have indicated that vaccination against COVID-19 elicits an effective immune response, manifested by the presence of anti-SARS-CoV-2 neutralizing antibodies in human milk. This finding suggests a potential capacity to confer passive protection to infants [13,14].

However, no studies on this topic have evaluated the percentage of antibodies generated by different vaccine technologies based on their potential neutralizing effects and COVID-19 variants. Therefore, the present study aims to determine the percentage and duration of neutralizing antibodies against the Omicron variant in human milk after vaccination against SARS-CoV-2, considering the three different vaccine technologies approved by the National Health Surveillance Agency (ANVISA) used in Brazil.

## 2. Materials and Methods

### 2.1. Study Design

This cross-sectional study was conducted at the Fernandes Figueira National Institute of Women, Children, and Adolescent Health (IFF) from April to October 2022. Breastfeeding women aged 18 or over living in Rio de Janeiro who had received a complete primary vaccination cycle with the COVID-19 vaccines available in Brazil (first and second doses of AstraZeneca, Pfizer, CoronaVac, or a single dose of Janssen), with or without booster doses, were considered eligible. Breastfeeding women with a milk volume of less than 10 mL and those who did not reach a blood volume of 5 mL were excluded.

After obtaining acceptance and the due signature of the informed consent form, properly trained researchers proceeded to apply a standardized and structured questionnaire exclusively developed for this research. This questionnaire covered sociodemographic aspects, information related to prenatal care, clinical data, nutritional status, and the date of administration of both the first and second dose (or single dose) and, when applicable, information about booster doses of the vaccines and the manufacturers of the vaccines received.

### 2.2. Data Collection

The milk was collected at the IFF’s Human Milk Bank (BLH). It was conducted by previously trained researchers or professionals from the sector, appropriately dressed in a lab coat, cap, mask, and disposable gloves. A Medela electric pump was used to collect 10 mL of milk. Manual milking was performed in cases of discomfort or inconvenience with using the pump or when women refused to use it.

The milk samples were then delivered to the High-Complexity Laboratory of IFF (LACIFF), where they were centrifuged at 2000 rpm at 4 °C for 25 min, and the supernatant was aliquoted into cryogenic vials and stored at −20 °C until use. Before processing, the breast milk samples were thawed and centrifuged at 2000 rpm for 15 min. The fat was removed, and the supernatant was transferred to a new tube. Centrifugation was repeated twice to ensure the removal of all fat cells.

### 2.3. Sample Analysis and Data Processing

The enzyme-linked immunosorbent assay (ELISA) method with a cPass™ SARS-CoV-2 Neutralization Antibody kit (GeneScript, Piscataway, NJ, USA) was employed to detect the percentage of antibodies with neutralizing potential for SARS-CoV-2 in breast milk samples. This test was designed to mimic the interaction of the virus with the host by direct protein–protein interaction in an enzyme-linked immunosorbent assay (ELISA) plate using the purified receptor-binding domain (RBD) of the Spike protein and the ACE2 host cell receptor. This particular interaction can then be inhibited/neutralized by neutralizing antibodies in the serum or milk of individuals, similar to what occurs in a conventional virus neutralization test. This test guarantees 100% specificity and 93% sensitivity for samples above the 30% inhibition cutoff (GeneScript). The assays were performed under the manufacturer’s instructions.

Milk samples were diluted in commercial dilution buffer provided by the kit in 96-well plates at a final volume of 100 µL. Dilutions of 10, 20, 50, or 100 times were applied to the different samples according to the need. Then, 100 µL of a solution containing the RBD domain of the original SARS-CoV-2 (Wuhan isolate) or the Omicron variant (B.1.1.529) conjugated to peroxidase (HRP) was added. The plate was incubated at 37 °C for 30 min. Subsequently, 100 µL of each well containing the sample and RBD-HRP was transferred to the test plate adsorbed with the human ACE2 molecule. The plates were incubated for 15 min at 37 °C, and the wells were washed four times with 300 µL of the kit’s washing solution and seventy times with 100 µL of the substrate 5,5′-tetramethyl-benzidine (TMB) added for incubation for 15 min in the dark at room temperature. The reaction was stopped by adding 50 µL of the kit’s “stop” solution and read at 450 nm. The calculation of the proportion of antibodies with neutralizing potential was then determined by the equation below using the kit’s negative control:

(1) $$\left(1-\left(Absorbance\;value\;of\;the\;sample\;÷\;Absorbance\;value\;of\;the\;negative\;control\right)\right)\times 100\%$$

Values below 30% were considered negative by the kit. This cutoff point is based on validation panels of sera from COVID-19 patients, which confirmed the presence of neutralizing antibodies by the 50% Reduction Neutralization Test (PRNT50) method and a panel of negative sera. The results are reported qualitatively as positive or negative for the presence of neutralizing antibodies. Statistical analyses were conducted using SPSS software, version 22, and R, version 4.0.3, with a significance level of 5% (*p* < 0.05) as a reference.

Categorical variables are described as absolute and percentage frequencies, while numerical variables are shown as median values and interquartile ranges (IQR). Normality tests, such as the Shapiro–Wilk or Kolmogorov–Smirnov tests, were performed to evaluate the data distribution. The Kaplan–Meier curve was adopted to identify the probabilities of the presence of antibodies with neutralizing potential over time, using the interval between the last dose of the vaccine received and the milk collection.

### 2.4. Ethical Aspects

The IFF Human Research Ethics Committee approved the study (CAAE: 52186521.2.0000.5269). Participation in the study was subject to signing a consent form, obtained freely and spontaneously after providing all clarifications pertinent to the main study. All postpartum women were informed during the reading that they could withdraw from the study at any stage. This study complies with the ethical principles of non-maleficence, beneficence, justice, and autonomy under Resolution N° 466/12 of the National Health Council.

## 3. Results

Sixty-six lactating women were eligible and included in the study. The mean age of the participants was 35 years (95% CI: 34.03–36.5). Regarding sociodemographic characteristics, most were white (71%), lived with a partner (93.9%), had incomplete or complete higher education (92.1%), and had attended at least six prenatal care appointments (99.5%). Regarding pre-gestational body mass index (BMI), most lactating women started pregnancy with an adequate weight (58.4%) (Table 1).

Regarding the frequency of SARS-CoV-2 infection prior to vaccination, 62.1% of the participants reported testing positive for COVID-19. Regarding the number of COVID-19 vaccine doses, 93.5% of the participants received three or more doses, and the Pfizer/BioNTech mRNA vaccine was the most commonly administered for both the first and booster doses (Table 2).

**Figure 1:**
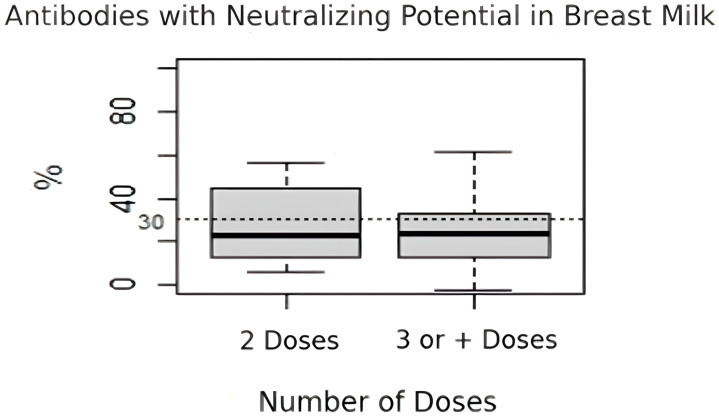
Percentage of antibodies with neutralizing potential in breast milk among women who received two doses of the vaccine and who received three or more doses.

Regarding the percentage of antibodies with neutralizing potential in breast milk, there was no significant difference between the women who received two doses of the vaccine (median = 22.9%, IQR: 12.8–44.9%) and those who received three or more doses (median = 23.5%, IQR: 12.8–33.3%) (*p* = 0.578) (Figure 1).

**Figure 2:**
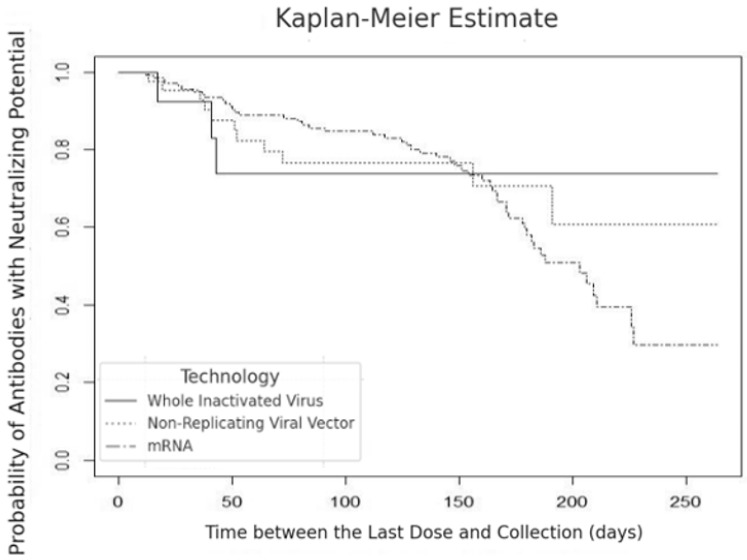
Time between last dose and human milk collection versus probability of antibodies with neutralizing potential in human milk across different vaccine technologies (inactivated virus, non-replicating viral vector, mRNA).

We identified a decline in the percentage of neutralizing antibodies in breast milk to 90% in 15 days, with a reduction to 75% at 50 days, remaining at that level for the subsequent 200 days for the inactivated virus technology (SinoVac/CoronaVac, Beijing, China/Serrana, Brazil). In the non-replicating viral vector technology (AstraZeneca, London, UK or Janssen-Johnson & Johnson, New Brunswick, NJ, USA), we observed a drop in the percentage of antibodies to approximately 75% between 50 and 100 days, with a further decline to 60% after 200 days of vaccination. In the mRNA technology (Pfizer/BioNTech, New York, NY, USA/Mainz, Germany), the percentage of antibodies with neutralizing potential decreased continuously to 45% up to 180 days (Figure 2).

The Kaplan–Meier curve analysis revealed the probability of antibodies with neutralizing potential in breast milk over 180 to 200 days after vaccination, considering different vaccine technologies (inactivated virus, non-replicating viral vector, and mRNA). Based on the curves, we observed that the probability of detecting neutralizing antibodies in breast milk reduced similarly over time, regardless of the vaccine technology adopted (Figure 2).

**Figure 3:**
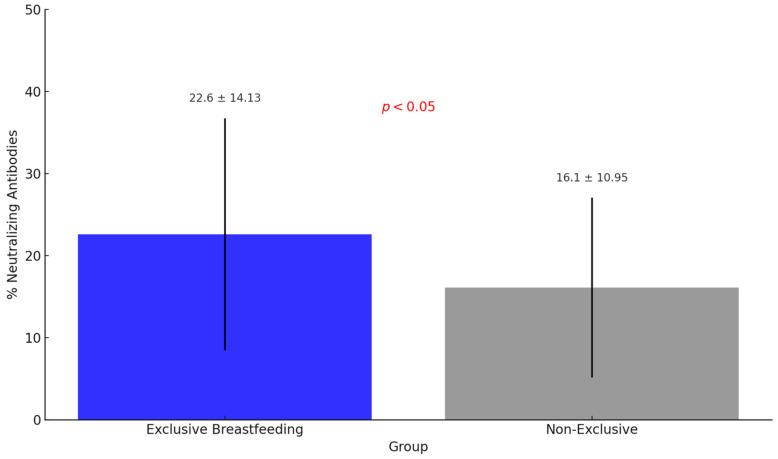
Comparison of neutralizing antibody levels against Omicron variant in breast milk between exclusive and non-exclusive breastfeeding mothers.

The statistical analysis showed no significant difference in the probability of antibodies with neutralizing potential between the different vaccine technologies (*p* = 0.900) (Figure 2). Furthermore, the percentage of neutralizing antibodies in breast milk from women who were exclusively breastfeeding was significantly higher (22.6% ± 14.13) than women not exclusively breastfeeding (16.1% ± 10.95) (*p* < 0.05) (Figure 3).

## 4. Discussion

The recommended exclusive breastfeeding for infants under six months of age, which comprises approximately 180 days, is an essential pillar of child health guidelines [7]. This practice plays a crucial role in infant nutrition. It has notable implications for their immunity due to the transfer of antibodies that help prevent the spread of viruses and serve as therapeutic agents for the rapid prevention and treatment of diseases such as SARS-CoV-2 [15].

This study’s results reinforce the importance of exclusive breastfeeding in women vaccinated against COVID-19, since the percentage of neutralizing antibodies in breast milk was significantly higher among women with exclusive breastfeeding than those without. Furthermore, we should highlight that in Brazil, vaccination against COVID-19 has not yet been approved for infants under six months of age under the guidelines of the National Immunization Program [16]. This finding reinforces the crucial role of exclusive breastfeeding in transferring protective antibodies to infants, significantly contributing to passive immunity against SARS-CoV-2 [17].

Corroborating these findings, Gray et al. (2021) showed that lactating mothers vaccinated with mRNA technology had high neutralizing antibody levels in their breast milk, which could potentially provide passive immunity to infants. The study suggested that exclusive breastfeeding may maximize the transfer of these antibodies [13]. Similarly, Jakuszko et al. (2021) found that exclusive breastfeeding can effectively provide infants with passive immunity, reinforcing this practice’s relevance, especially during viral outbreaks and epidemics [17].

Furthermore, the present study evidenced a high percentage of neutralizing antibodies in breast milk in the first 180 days after vaccination, regardless of the vaccine technology used and the number of doses administered. This result was corroborated by the cohort study conducted by Yeo et al. (2022), which aimed to evaluate the presence and persistence of neutralizing antibodies in the breast milk of lactating women vaccinated against SARS-CoV-2. This study involved 35 lactating women who received two doses of mRNA-based COVID-19 vaccines, specifically BNT162b2 (Pfizer-BioNTech, New York, NY, USA/Mainz, Germany) and mRNA-1273 (Moderna, Cambridge, MA, USA), with a 21-day interval between doses. The results of this study confirmed the significant presence of neutralizing antibodies in breast milk during the first 180 days after vaccination, regardless of the vaccine technology or the number of doses administered. Furthermore, these antibodies persisted robustly over time, showing that mRNA-based vaccination offered prolonged protection to infants through passive immunity conferred by breast milk [18].

Consolidating the evidence that breastfeeding plays a crucial role in the transfer of passive immunity to infants, the cohort study conducted by Narayanaswamy et al. (2022) [19] revealed the presence of neutralizing antibodies in approximately 30% of infant stool samples, strengthening the passage of active antibodies from breast milk to the newborn’s gastrointestinal tract. These findings underscore the efficacy of breastfeeding as a form of immunological protection, evidencing that infants receive and retain neutralizing antibodies that confer passive immunity against SARS-CoV-2. Identifying these antibodies in infant feces is direct evidence of the successful transfer of immunity from mother to infant, consolidating the importance of breastfeeding in protecting newborns against viral infections, including SARS-CoV-2 [19].

The vaccines evaluated in this study were approved in Brazil as part of the national strategy to combat the COVID-19 pandemic coordinated by the National Immunization Program (PNI). The vaccines analyzed comprise different technological platforms, including mRNA vaccines (Pfizer/BioNTech), non-replicating viral vectors (AstraZeneca and Janssen), and inactivated virus (CoronaVac). Initially, the PNI prioritized vaccinating individuals most vulnerable to severe forms of the disease, such as older adults, people with comorbidities, health professionals, pregnant women, and lactating women, highlighting the relevance of immunizing these groups [16].

This study showed that regardless of the vaccine technology used, all types were effective in inducing a robust immune response with the production of neutralizing antibodies in human milk, evidencing a consistent response throughout the period evaluated. These findings strengthen the effectiveness of the different vaccine platforms in promoting passive immunity in infants. This finding is particularly relevant, since most previous studies have evaluated only one type of technology, predominantly mRNA, without comparatively exploring the performance of other platforms [9,13]. Also, most studies on this topic have not evaluated the effect of vaccines considering the variants. This evaluation of the three types of technology approved by ANVISA in Brazil contributes to a more comprehensive understanding of the effectiveness of transferring passive immunity to infants.

Although the WHO declared the end of the public health emergency of international concern on 5 May 2023, this does not mean that COVID-19 has ceased to be a health risk. The global spread of the disease is still referred to as a pandemic, suggesting that it is time for countries to move from an emergency mode to managing COVID-19 alongside other infectious diseases [20]. Although the pandemic’s peak has passed, its impact will be significant and long-lasting. The SARS-CoV-2 virus that caused the pandemic still kills one person every three minutes, while many survivors suffer the debilitating effects of long COVID for months [21]. Since immunization against COVID-19 has not been recommended for children under six months of age in Brazil under the PNI guidelines [16], this study’s results highlight the importance of exclusive breastfeeding as an essential strategy to provide immunological protection to infants, underscoring the need to promote and support this practice as a vital measure for child health, especially in pandemic contexts.

One limitation of this study is related to the profile of the population analyzed, which was primarily composed of highly educated women with adequate access to health services and who underwent regular prenatal care follow-up. This profile may have contributed to greater adherence to the complete vaccination cycle, which does not necessarily reflect the reality of other populations. Therefore, the results should be interpreted with caution when generalizing to groups with different socioeconomic conditions and access to healthcare that may have lower vaccination coverage rates or inadequate monitoring during pregnancy, since, according to Shakibazadeh et al. (2022), less-educated pregnant women have lower rates of adherence to vaccination against COVID-19 [22].

Finally, we should underscore that although the vaccines were initially developed for the original strain of SARS-CoV-2, the emergence of variants of concern, such as Omicron, which has multiple mutations in the Spike protein, can alter the neutralization capacity of the antibodies generated [23]. Although the data from this study point to persistent neutralizing antibodies over time, their efficacy against these emerging variants requires further investigation. Thus, our findings reinforce the need for continued studies to evaluate the impact of viral mutations on passive immunity transmitted to infants, especially considering the different vaccine technologies employed.

## 5. Conclusions

Neutralizing antibodies are crucial in combating SARS-CoV-2 and can be induced by vaccination and infection. This study revealed a significant increase in the levels of neutralizing antibodies in the breast milk of mothers who exclusively breastfed their infants. These findings align with other research confirming the transfer of these antibodies to breast milk, highlighting the importance of exclusive breastfeeding in the first six months of life.

This interconnection between the period of exclusive breastfeeding and the persistent neutralizing antibodies in milk strengthens breastfeeding’s contribution to the active immunization of infants regarding COVID-19, given that immunization against COVID-19 in Brazil was not recommended for children under six months of age under the PNI guidelines.

Finally, understanding the immune response, especially the presence and persistence of neutralizing antibodies, is crucial in advancing scientific knowledge and formulating effective public health strategies. The lack of studies in this area is a significant gap that needs to be filled to strengthen the capacity to respond to various pathogens. Therefore, promoting and supporting research that investigates the presence of neutralizing antibodies for different pathogens is essential to broaden the understanding of immune defense mechanisms and, consequently, improve approaches to preventing and controlling infectious diseases.

## Figures and Tables

**Table 1:** Maternal characteristics and prenatal care data.

Variable	Mean	Standard Deviation
Age (mean)	35.09	5.26
How many months of pregnancy did you start prenatal care?	1.61	0.63
How many prenatal consultations did you have during this pregnancy?	11.43	2.96
Pre-pregnancy BMI (mean)	24.06	4.12
Days between birth and sample collection (mean)	284.31	231.62

**Table 2:** Distribution of maternal characteristics, health conditions, and COVID-19.

Category	Frequency	Percentage
Lives with partner		
No	5	7.6%
Yes	61	92.4%
Race/color		
White	39	59.1%
Black	7	10.6%
Brown/Asian/Indigenous	20	30.3%
Education		
Incomplete/Complete Elementary School	1	1.5%
Incomplete/Complete High School	7	10.6%
Incomplete/Complete Higher Education	58	87.9%
Works		
No	15	22.7%
Yes	51	77.3%
Had prenatal care in this pregnancy		
No	1	1.5%
Yes	65	98.5%
Hypertension in this pregnancy		
No	61	92.4%
Yes	5	7.6%
Diabetes in this pregnancy		
No	59	89.4%
Yes	7	10.6%
Hypothyroidism or hyperthyroidism in this pregnancy		
No	16	89.4%
Yes	7	10.6%
BMI (pre-pregnancy)		
Underweight	2	3.0%
Normal	38	57.6%
Overweight	20	30.3%
Obesity	6	9.1%
Weight gain		
Below	10	15.15
Adequate	21	31.82
Above	35	53.03
COVID-19 test result		
Positive	41	62.12
Negative	25	37.88
Vaccine		
CoronaVac	10	15.15
AstraZeneca (Fiocruz)	17	25.76
Pfizer	37	56.06
Janssen	2	3.03
Number of doses		
2	5	7.58
3 ou +	61	92.42

## Data Availability

The raw data supporting the conclusions of this article will be made available by the authors on request.
